# Perceived Risk and Protection From Infection and Depressive Symptoms Among Healthcare Workers in Mainland China and Hong Kong During COVID-19

**DOI:** 10.3389/fpsyt.2020.00686

**Published:** 2020-07-15

**Authors:** Simon Ching Lam, Teresa Arora, Ian Grey, Lorna Kwai Ping Suen, Emma Yun-zhi Huang, Daofan Li, Kin Bong Hubert Lam

**Affiliations:** ^1^ Squina International Centre for Infection Control, School of Nursing, The Hong Kong Polytechnic University, Hung Hom, Hong Kong; ^2^ Department of Psychology, College of Natural & Health Sciences, Zayed University, Abu Dhabi, United Arab Emirates; ^3^ School of Arts & Sciences, Lebanese American University, Beirut, Lebanon; ^4^ Division of Pre-school Education, Zhongshan Polytechnic, Zhongshan, China; ^5^ Special Geriatric Committee, Zhongshan Medical Association, Zhongshan, China; ^6^ Nuffield Department of Population Health, University of Oxford, Oxford, United Kingdom

**Keywords:** COVID-19, depression, healthcare workers, perceived vulnerability, personal protective equipment

## Abstract

Psychological health among healthcare workers (HCWs) has become a major concern since the COVID-19 outbreak. HCWs perceived risks of contracting COVID-19, in relation to depression were investigated. It was hypothesized that perceived high risk of contracting COVID-19 (close contact with cases, inadequate provision of personal protective equipment, insufficient infection control training, and presence of symptoms) would be significant predictors of depression. Our cross-sectional survey was completed by HCWs across three regions (Hubei, Guangdong, Hong Kong) between March 9 to April 9 2020 using convenience sampling. Depression was assessed using the 9-item Patient Health Questionnaire (PHQ-9). Prevalence of depression was 50.4% (95% CI: 44.5-56.2), 15.1% (10.1-21.9) and 12.9% (10.3-16.2) for HCWs in Hong Kong, Hubei and Guangdong, respectively. The strongest significant risk factors for depression, after adjustment, were HCWs who reported the greatest extent of feeling susceptible to contracting COVID-19 and those who reported the greatest difficulty obtaining face masks. HCWs whose family/peers greatly encouraged face mask use had lower prevalence of depression. Access to adequate supplies of personal protective equipment is essential for the psychological health of HCWs working in stressful environments, through potentially easing their perceptions of vulnerability to COVID-19.

## Introduction

In the immediate aftermath of the outbreak of COVID-19 in Wuhan, Hubei Province, China, a number of tertiary hospitals in adjacent regions moved quickly to establish psychological intervention programs to support healthcare workers (HCWs) working with infected, and potentially infected patients (1, 2). This reflects the recognition of the centrality of maintaining optimal psychological functioning among HCWs for effective healthcare service delivery, and an implicit recognition that HCWs are adversely affected by both physical and psychological stresses (3). While the effectiveness of these programs, which include the provision of online courses on dealing with common psychological problems, hotlines to provide psychological assistance, and group-based activities designed to reduce stress,(1) has yet to be evaluated, it has been reported that staff are reluctant to engage with such services (1).

The question remains as to how it can be best achieved to support the psychological well-being of HCWs working in high-risk environments under high-pressure and anti-pandemic situations (4). Identification of specific psychological difficulties and their risk factors are required for a multi-faceted and nuanced approach to developing effective evidence-based support (5, 6). Individual responses may vary as a function of intrapersonal risk perception and resilience, and workplace environmental and organizational factors including training, availability and use of personal protective equipment (PPE) (e.g., protective face wear, gloves, aprons, gowns),(7) all of which may explain the psychological effects frontline HCWs experience during COVID-19 (3).

The majority of HCWs report the protection of one’s own physical health as a primary concern in pandemic situations (6). Indeed, HCWs are expected to have adequate workplace protection measures (7). The limited availability of PPE due to global shortage and perceived risk of contracting the disease when exposed to infected patients may potentially underscore the onset of mental health symptoms. Confidence in infection-control measures, such as the effectiveness of face masks may reduce risk perception and may also mitigate and facilitate an adaptive stress response. However, what remains largely absent from the literature to date, is an examination of actual pandemic situations and if perceived risk (vulnerability and fear of contracting) and mitigations (effectiveness of face masks and knowledge of COVID-19) are associated with mental health difficulties, such as depression, in physicians and nurses. Here we report findings from an online survey of HCWs across three regions of China.

## Methods

### Study Design and Population

We conducted an internet-based survey among HCWs in Hubei (mainly Wuhan), Guangdong (from the cities of Foshan, Shenzhen, Zhongshan, Zhuhai) provinces of Mainland China, and Hong Kong between March 9, 2020, and April 9, 2020, using a convenience sampling method. HCWs from a variety of practice settings, including hospitals and clinics were invited using various online platforms (e.g., discussion boards of societies of healthcare professionals, Facebook, Instagram, WeChat). For HCWs who accessed the link from the online platforms, study-related information was presented and respondents were then asked to indicate their consent preference (“agree” or “not agree”). Those who provided consent were then presented with the survey questionnaire (described below). Participation was voluntary and anonymous. Of the 1566 HCWs who accessed the link, 42.5% (146/343) in Hubei, 65.4% (510/780) in Guangdong and 62.3% (276/443) in Hong Kong agreed to participate. There were no exclusion criteria for the study and data from all participants who provided positive consent were included. The study was approved by the Human Subjects Ethics Sub-committee of the Special Geriatric Committee of Zhongshan Medical Association (SGCZSMA20200306) and was carried out in accordance with the Declaration of Helsinki.

### Questionnaire

The 35-item questionnaire, administered in Chinese, was comprised of four sections (see **Supplementary Material**). Section 1 enquired about demographics and their profession. Section 2 enquired about the provision of training and face masks/respirators in their hospital/clinic for the COVID-19 epidemic. Section 3 explored the factors leading to or associated with face mask/respirator use, using the Health Belief Model framework, modified from previous studies (8). Section 4 was the nine-item Patient Health Questionnaire-9 (PHQ-9), used for screening and measuring the severity of depressive symptoms (9).

### Depressive Symptoms

The outcome of interest was the presence of depressive symptoms, as measured by the PHQ-9, which scores each of the nine diagnostic criteria of the Diagnostic and Statistical Manual of Mental Disorders, 4^th^ Edition (DSM-IV) for depression from 0 (not at all) to 3 (nearly every day) over the previous two weeks (10). With a cut-off score of 9, PHQ-9 had a sensitivity of 80% and a specificity of 92% for diagnosing major depression in the Hong Kong Chinese population (11). For this study, those who had scores of 15-27 were considered to have moderate/severe depression.

### Exposure

Due to the ongoing nature of the COVID-19 outbreak, we considered four exposures associated with COVID-19, *a priori*, as potential risk factors for depression among HCWs over the previous two weeks. These included: (i) close contact with confirmed or suspected COVID-19 cases; (ii) inadequate provision of personal protective equipment; (iii) insufficient infection control training; and (iv) having symptoms recently similar to those manifested in a COVID-19 infection.

Participants also rated on a four-point scale (not at all/to a small extent/to a moderate extent/to a very great extent) the degree (i) they felt susceptible to COVID-19 infection; (ii) the fear of contracting coronavirus; (iii) face masks could prevent them from contracting coronavirus; (iv) difficulty obtaining face masks; (v) family/peers encouraged them to wear face masks; and (vi) if their knowledge about COVID-19 was adequate.

### Statistical Analysis

We computed the mean PHQ-9 scores and the prevalence of PHQ-9-positive depressive symptoms, both with 95% confidence interval (CI) and compared them across the regions (Hong Kong versus Guangdong and Hong Kong versus Hubei) using Cohen’s d (for PHQ-9 scores) and Cohen’s w (for the prevalence). We estimated the prevalence of depressive symptoms according to the number of risk factors they were exposed to (0, 1, 2, ≥3), and the health belief related to face mask use. We investigated the association between health belief and depression by building two logistic models, which yielded adjusted odds ratios (ORs) and 95% CI. In model 1, we entered the six health belief variables separately, each adjusting for sex, educational level, marital status, location, profession, ward/unit, year of hospital work experience (continuous), and the presence of *a priori* risk factors (0, 1, 2, ≥3). In model 2 all health belief variables were mutually adjusted for, in addition to those already entered in model 1. Because of the small number of responses in the “not at all” category, we combined them with “to a small extent” in all variables and considered the new group as the reference category. Based on the regression output, we estimated the prevalence of depression according to health belief variables, stratifying for location.

All statistical analyses were performed using Stata version 13.0 (StataCorp LP). P-values were 2-tailed and those <0.05 were considered statistically significant.

## Results

The sample characteristics of 932 HCWs are presented in **Table 1**, stratified by region. There were more females than males across each location. The percentage of HCWs in Hong Kong reporting inadequate provision of PPE was high (77.5%), and the majority of these HCWs also reported insufficient infection-control training (72.1%). Furthermore, 15.2% of HCWs in Hong Kong claimed they were experiencing COVID-like symptoms, greater than those in Hubei (4.1%) and Guangdong (3.3%).

**Table 1:** Characteristics of 932 healthcare workers from Mainland China and Hong Kong.

	Guangdong(n=510)	Hubei(n=146)	Hong Kong(n=276)
Sex			
Male	129 (25.3)	54 (37.0)	48 (17.4)
Female	381 (74.7)	92 (63.0)	228 (82.6)
Married	390 (76.5)	88 (60.3)	96 (35.3)
Educational level			
College or below	106 (20.8)	32 (21.9)	106 (38.8)
Undergraduate or above	404 (79.2)	114 (78.1)	167 (61.2)
Profession			
Nurses	233 (45.7)	89 (61.0)	258 (93.5)
Physicians	208 (40.8)	52 (35.6)	3 (1.1)
Other	69 (13.5)	5 (3.4)	15 (5.4)
Ward/Unit			
COVID	1 (0.2)	9 (6.2)	0 (0.0)
Accident and emergency	30 (5.9)	13 (8.9)	18 (6.5)
Intensive care	25 (4.9)	63 (43.2)	8 (2.9)
Isolation ward	3 (0.6)	0 (0.0)	10 (3.6)
Infection control	3 (0.6)	12 (8.2)	4 (1.5)
Respiratory	21 (4.1)	11 (7.5)	4 (1.5)
Medical	67 (13.1)	16 (11.0)	64 (23.2)
Surgical	49 (9.6)	4 (2.7)	48 (17.4)
Maternity and pediatric	95 (18.6)	2 (1.4)	26 (9.4)
Community and out-patient clinic	48 (9.4)	4 (2.7)	17 (6.2)
Other	168 (32.9)	12 (8.2)	77 (27.9)
Work experience in hospital; years	12 (6-20)	10 (6-15)	5 (2-10)
Risk factors			
Close contact with confirmed or suspected COVID-19 cases	112 (22.0)	112 (76.7)	135 (48.9)
Inadequate provision of personal protective equipment	168 (32.9)	35 (24.0)	214 (77.5)
Insufficient infection control training	28 (5.5)	4 (2.7)	199 (72.1)
Presence of COVID-19-like symptoms	17 (3.3)	6 (4.1)	42 (15.2)

On average, Hong Kong HCWs had a higher PHQ-9 score (*mean [SD]:* 10.5 [6.4]) compared to those in Hubei (5.4 [4.6]; Cohen’s d=0.86; p<0.001) and Guangdong (4.6 [4.8]; Cohen’s d=1.09; p<0.001). The differences in PHQ-9 scores across regions were smaller when controlling for demographic and organizational factors (**Table 2**). There was no meaningful difference in the PHQ-9 scores between nurses and physicians within each region (see **Supplementary Table 1**). More than half of the respondents in Hong Kong met the criteria for depression (50.4%), which was considerably higher than those in Hubei (15.1%; Cohen’s w=0.35; p<0.001) and Guangdong (12.9%; Cohen’s w=0.41; p<0.001). After adjusting for the demographic and organizational factors, the prevalence of depression among HCWs in Hong Kong was still considerably higher (30.0%, 95% CI: 21.1%-38.9%) than that in Guangdong (15.9%, 12.0%-19.9%; p=0.008) and Hubei (16.5%, 8.0%-25.0%; p=0.051) (**Table 2**). There was a dose-response relationship between the number of *a priori* risk factors (close contact with COVID-19 cases; inadequate PPE provision; insufficient infection control training; and having COVID-19-like symptoms) and prevalence of depression. For example, in Hong Kong, 64% of the HCWs with 3 or 4 risk factors had PHQ-9-positive depression compared to 8.3% among those none of the risk factors (see **Supplementary Table 2**).

**Table 2:** PHQ-9 score and screen-detected positive depression among 932 Chinese healthcare workers during COVID-19.

	**Guangdong** **(n=510)**	**Hubei** **(n=146)**	**Hong Kong** **(n=276)**
PHQ-9 score			
Mean (SD)	4.6 (4.8)	5.4 (4.6)	10.5 (6.4)
Effect size^#^	1.09	0.86	Reference
p-value	<0.001	<0.001	
Adjusted* mean (95% CI)	4.9 (4.2-5.5)	5.7 (4.5-6.9)	7.5 (6.6-8.5)
Difference; mean (95% CI)	2.7 (1.3-4.0)	1.8 (0.2-3.5)	Reference
p-value	<0.001	0.029	
PHQ-9 positive (score ≥9)			
n	66	22	139
Prevalence; % (95% CI)	12.9 (10.3-16.2)	15.1 (10.1-21.9)	50.4 (44.5-56.2)
Effect size^	0.41	0.35	Reference
p-value	<0.001	<0.001	
Adjusted* prevalence; % (95% CI)	15.9 (12.0-19.9)	16.5 (8.0-25.0)	30.0 (21.1-38.9)
Difference; % (95% CI)	14.1 (0.4-24.5)	13.5 (-0.01-27.1)	Reference
p-value	0.008	0.051	
Moderately severe/severe depression (score ≥15)			
n	23	7	74
Prevalence; %	4.5 (3.0-6.7)	4.8 (2.3-9.7)	26.8 (21.9-32.4)
Effect size^	0.32	0.27	
p-value	<0.001	<0.001	
Adjusted* prevalence; % (95% CI)	5.7 (3.1-8.3)	4.8 (-0.1-9.7)	8.0 (3.3-12.7)
Difference; % (95% CI)	3.2 (-3.7-10.2)	2.3 (-3.0-7.6)	Reference
p-value	0.402	0.364	

*Adjusted for sex, educational level, marital status, location, profession, ward/unit, work experience, close contact with confirmed or suspected COVID-19 cases, personal protective equipment provision, infection control training, and presence of COVID-19-like symptoms.

^#^Cohen’s d.

^Cohen’s w.

Data surrounding health beliefs and personal views of face mask use during COVID-19 in relation to depression, by location, are presented in **Table 3**. Of note, 80% of those in Hong Kong who felt susceptible to COVID-19 to a very great extent met the criteria for depression. In the same group, 64.7% of those who felt very fearful of contracting COVID-19 screened positive for depression. The prevalence of depression appeared to higher with the increasing the level of perceived vulnerability and fear and frustration of not being able to obtain face masks (see **Supplementary Table 3**).

**Table 3:** Prevalence of depression according to health beliefs among 932 Chinese healthcare workers during COVID-19.

	**Guangdong**	**Hubei**	**Hong Kong**
*Feeling susceptible to COVID-19 infection*			
Not at all/to a small extent			
n	32/363	12/106	34/103
PHQ-9 positive; % (95% CI)	8.8 (6.3-12.2)	11.3 (6.5-19.0)	33.0 (24.6-42.7)
To a very great extent			
n	11/48	1/15	36/45
PHQ-9 positive; % (95% CI)	22.9 (13.1-37.0)	6.7 (0.9-37.3)	80.0 (65.6-89.4)
*Fearful of contracting COVID-19*			
Not at all/to a small extent			
n	31/340	15/104	15/61
PHQ-9 positive; % (95% CI)	9.1 (6.5-12.7)	14.4 (8.8-22.7)	24.6 (15.3-37.0)
To a very great extent			
n	14/65	2/10	66/102
PHQ-9 positive; % (95% CI)	21.5 (13.1-33.3)	20.0 (4.6-56.5)	64.7 (54.9-73.4)
*Wearing face mask could prevent contracting COVID-19*			
Not at all/to a small extent			
n	5/34	1/9	7/17
PHQ-9 positive; % (95% CI)	14.7 (6.2-31.2)	11.1 (1.3-53.6)	41.2 (20.5-65.6)
To a very great extent			
n	32/280	8/95	89/180
PHQ-9 positive; % (95% CI)	11.4 (8.2-15.7)	8.4 (4.2-16.1)	49.4 (42.2-56.8)
*Difficult to get face masks*			
Not at all/to a small extent			
n	31/308	14/121	31/98
PHQ-9 positive; % (95% CI)	10.1 (7.2-14.0)	11.6 (6.9-18.7)	31.6 (23.1-41.6)
To a very great extent			
n	14/76	2/5	53/76
PHQ-9 positive; % (95% CI)	18.4 (11.2-28.8)	40.0 (8.1-83.4)	69.7 (58.4-79.1)
*Encouraged by family and peers to wear face mask*			
Not at all/to a small extent			
n	4/27	3/12	13/18
PHQ-9 positive; % (95% CI)	14.8 (5.6-34.0)	25.0 (7.8-56.9)	72.2 (47.2-88.3)
To a very great extent			
n	40/351	12/107	100/201
PHQ-9 positive; % (95% CI)	11.4 (8.5-15.2)	11.2 (6.4-18.8)	49.8 (42.8-56.7)
*Having adequate knowledge about COVID-19*			
Not at all/to a small extent			
n	4/30	1/5	32/56
PHQ-9 positive; % (95% CI)	3.3 (0.4-20.8)	20.0 (2.1-74.7)	57.1 (43.8-69.5)
To a very great extent			
n	25/213	7/79	30/65
PHQ-9 positive; % (95% CI)	11.7 (8.0-16.8)	8.9 (4.2-17.6)	46.2 (34.3-58.4)

After adjustment for a range of potential confounding factors, those with *a priori* risk factors had 2 to 4 times the odds of screen-detected depressive symptoms compared to those without (*OR*=2.24, *95% CI*: 1.29-3.92 for 1 risk factor; 2.36, 1.25-4.42 for 2 risk factors; and 3.61, 1.65-7.91 for 3 or 4 risk factors). Among the health beliefs, feeling susceptible and fearful of contracting COVID-19, as well as difficulty obtaining face masks are associated with higher risk of depression (**Table 4**). Those having the strongest feeling of vulnerability and fear of contracting COVID-19 and those who found it extremely difficult to obtain face masks had approximately three times the odds of being screened positive for depressive symptoms, compared to those who did not have such feeling or problem. Interestingly, a strong belief that face mask could be protective was not associated with depression (*OR*=1.00, *95% CI:* 0.42-2.36) while encouragement by family and peers to wear a face mask to a great extent appeared to be a protective factor against depression, after adjustment (*OR*=0.45, *95% CI:* 0.23-0.88). The estimated prevalence of depression according to the various health beliefs are presented in **Supplementary Table 3**.

**Table 4:** Logistic regression analysis of health beliefs as predictors of PHQ-9 screened positive depression among 932 Chinese healthcare workers.

	Model 1*		Model 2#	
	OR (95% CI)	P-trend	OR (95% CI)	P-trend
*Feeling susceptible to COVID-19 infection*				
Not at all/to a small extent	1.00	<0.001	1.00	<0.001
To a moderate extent	2.20 (1.45-3.34)		1.64 (1.03-2.59)	
To a very great extent	3.51 (2.14-5.74)		2.65 (1.55-4.54)	
*Fearful of contracting COVID-19*				
Not at all/to a small extent	1.00	<0.001	1.00	0.006
To a moderate extent	1.95 (1.27-3.00)		1.68 (1.04-2.72)	
To a very great extent	2.94 (1.84-4.70)		1.98 (1.19-3.29)	
*Wearing face mask could prevent contracting COVID-19*				
Not at all/to a small extent	1.00	0.049	1.00	0.221
To a moderate extent	1.50 (0.68-3.29)		1.50 (0.63-3.56)	
To a very great extent	0.89 (0.41-1.91)		1.00 (0.42-2.36)	
*Difficult to get face masks*				
Not at all/to a small extent	1.00	<0.001	1.00	<0.001
To a moderate extent	2.06 (1.35-3.14)		1.90 (1.22-1.98)	
To a very great extent	2.67 (1.66-4.28)		2.27 (1.39-3.71)	
*Encouraged by family and peers to wear face mask*				
Not at all/to a small extent	1.00	0.027	1.00	0.030
To a moderate extent	0.57 (0.29-1.13)		0.56 (0.27-1.14)	
To a very great extent	0.48 (0.26-0.87)		0.45 (0.23-0.88)	
*Having adequate knowledge about COVID-19*				
Not at all/to a small extent	1.00	0.085	1.00	0.367
To a moderate extent	1.09 (0.66-1.81)		1.23 (0.72-2.10)	
To a very great extent	0.71 (0.40-1.26)		0.88 (0.44-1.62)	

*Adjusted for sex, educational level, marital status, location, profession, ward/unit, work experience, and number of risk factors.

^#^Model 1 plus mutual adjustment of health belief variables.

## Discussion

To our knowledge, we are the first group to report the mental health status, specifically depression, among a large sample of HCWs across both the pandemic and non-pandemic regions of China as well as Hong Kong and to examine the relevance of perceived risk and protection from infection in relation to depression. We found that the prevalence of self-reported depression was considerably higher among HCWs in Hong Kong, compared to Guangdong and Hubei (where COVID-19 was first discovered). The observations in Guangdong and Hubei are similar to those in other surrounding Asian countries during the COVID-19 outbreak (12). More than a quarter of HCWs in Hong Kong reported symptoms indicative of moderately severe or severe depression. It is important to note that before the COVID-19 outbreak in Hong Kong, the prevalence of depression among general population in October 2019, during social crisis, was between 11.2% (13). However, findings from a cross-sectional study that were reported in a local newspaper, stated that depression was 18.5% (14). Social unrest is therefore likely to have influenced the prevalence of depression during the COVID-19 outbreak among HCWs in our study. Not surprisingly, among these 932 HCWs the feeling of susceptible to and being fearful of contracting the virus were strongly associated with depression. However, what was emerged as a significant predictor among respondents was reporting great difficulty in obtaining face masks in particular. In line with his, our findings also highlighted the encouragement of face mask use from family and peers as inversely related to depression.

While on first inspection, it may appear paradoxical that HCWs in Hubei had low rates of depression compared to other regions, this pattern of differences in depressive symptoms according to location may be attributable to the timing of survey distribution. It is important to note that the pandemic in Hubei, where the virus started in late 2019, authorities had almost gained control of the outbreak at the time of survey administration, which may explain the lower levels of depressive symptoms observed in Mainland China, including Hubei. However, by this time, the number of diagnosed COVID-19 cases rose sharply in Hong Kong (from 116 on March 9 to 974 on April 9), which may explain the higher prevalence of depressive symptoms among its HCWs. Although the majority of the responders in Hong Kong were nurses (93.5%), previous research has identified that rates of both depression and anxiety are typically higher in nurses compared to other healthcare workers (3). This observation may be reflected in the current study, thereby contributing to this increased prevalence. Perhaps of greater concern is the higher levels of concern expressed by Hong Kong HCWs expressing in relation to the inadequate provision of PPE (77.5% in Hong Kong versus 24.0% in Hubei and 32.9% in Guangdong), which we will now discuss.

COVID-19 is transmitted by person-to-person contact or droplet transmission of large respiratory particles that can travel approximately one-meter from the infected individual (15). The importance of adequate availability of PPE for staff in hospital settings amidst the COVID-19 outbreak has been documented (16). PPE, which was once ubiquitous in clinical settings, is now sparse across multiple locations due to the influx of hospitalized COVID-19 patients and also a rapid uptake of their use among the general population (17, 18). If HCWs are not suitably protected with PPE, or have inadequate supplies, then this unequivocally jeopardizes the physical health by increasing the actual and perceived level of subsequent risk of infection among HCWs who work with confirmed or suspected cases (19).

While the focus of previous research since the COVID-19 outbreak has been on PPE in relation to the physical health of HCWs (contracting the virus), our findings suggest that adequate availability of PPE may influence risk perception, as highlighted by previous research that risk perceptions of susceptibility and severity of infection could predict preventative behaviors such as face mask purchase and use during infectious disease pandemics (20, 21). Thus, limited availability of face masks is likely to have heightened perceived risk and the fear of becoming infected with COVID-19 in our sample, particularly if the HCWs were in direct, close contact with COVID-19 patients. This increased perception of risk and susceptibility may be further heightened by the number of dependents (children, elderly) associated with the HCWs, though we did not obtain this data in our study.

There is a reciprocal relationship between state anxiety and increased threat perception (22). Specifically, cognitive biases, when applied to the processing of threat, increase the level of state anxiety. Elevated state anxiety, in turn, amplifies or exaggerates cognitive biases. As a result, cognitive behavior therapy (CBT) may be helpful for improving mental health outcomes during the COVID-19 pandemic, given that it is a well-evidenced treatment for psychological disorders (23). However, the evidence for CBT as an intervention in HCWs remains preliminary (24). Larger trials that are specifically tailored to the COVID-19 scenario are therefore needed. As such, pre-existing cognitive vulnerabilities, combined with insufficient PPE (exemplified by the difficulty in obtaining face masks), may result in elevated symptomatology in some HCWs. This may partially why only a proportion of HCWs manifest with symptoms of depression and anxiety (25). In addition, one of the driving factors behind the heightened risk of depression which we observed beyond inadequate PPE, was perceived susceptibility and fear of contracting COVID-19. This latter finding resonates strongly with a cognitive bias model of understanding psychopathology (22). In line with our findings, another recently conducted survey among HCWs in Hong Kong revealed that 45.2% reported being concerned about adequate PPE and that 19.6% were worried about contracting COVID-19 (2). Although the authors did not link the two factors, they did report a total of 49.3% who met the criteria for depression (34.8% with mild and 14.5% with moderate) based on the PHQ-9, the same tool that we employed in our study.

The only protective factor that we observed in relation to depressive symptoms across our total sample was that of family and peer encouragement for the application of face masks. This may be seen as an act of solidarity, as the encouragement could imply the help to procure of face masks even though they had been not been provided in sufficient quantities in the hospital and were very difficult to be obtained from the market. Our observation is consistent with another study that demonstrated significantly lower depression prevalence among those practicing good personal hygiene techniques such as face mask use and regular hand washing (26). This is further consistent with previous research that shows how an individual’s social support networks is a key factor for mental health and wellbeing (27). In particular, social support networks have been specifically linked to depression levels rather than other psychological outcomes. Thus, meaningful social relationships and support are likely to play a pivotal role in the mental health and wellbeing of HCWs working in high risk environments during pandemics such as COVID-19.

We acknowledge some study limitations. First, our study relied on a convenience sample thus non-response bias is highly likely and may not be inferred to the whole HCW population. It is possible that the a considerable proportion of the HCWs who participated in this survey might want to use the opportunity to express their discontent towards the authority as there continued to be a chronic short supply of PPE in hospitals. Nevertheless, the same pattern emerged from all three regions suggesting this was not unique in Hong Kong. Second, all data are subjective but it is important to note that beliefs cannot be objectively ascertained, although these may be subject to biases including social desirability and recall, with the latter being a particularly important consideration in cognitively vulnerable individuals. Third, we did not obtain any data pertaining to age of our participants, which may be a potential confounding factor of the relationships assessed, although we have used number of years of experience as proxy. Fourth, due to the cross-sectional study design, we are unable to determine cause-effect relationships. Finally, the focus of our study was depression but we acknowledge that other psychological outcomes are also important although we did not obtain data on aspects such as anxiety and stress.

In conclusion, it is possible that waves of depressive symptoms may be observed in HCWs across COVID-19-affected countries when cases are peaking. It is essential that PPE demand is met to minimize and protect the mental and physical health of those who are working tirelessly to control the pandemic. The adverse consequences of insufficient physical protection (such as PPE provision and training on infection control) while having close contact with patients are likely to leave HCWs with higher perceived levels of risk in terms of fear and susceptibility of COVID-19, which manifest in depression. Psychological services should be provided to all HCWs, and social support from family members and peers are also fundamental to the psychological health of HCWs.

## Data Availability Statement

The datasets presented in this article are not readily available because restrictions imposed by the regulatory authorities. Requests to access the datasets should be directed to SL, simon.c.lam@polyu.edu.hk.

## Ethics Statement

The studies involving human participants were reviewed and approved by Human Subjects Ethics Sub-committee of the Special Geriatric Committee of Zhongshan Medical Association. Written informed consent for participation was not required for this study in accordance with the national legislation and the institutional requirements.

## Author Contributions

SL: Conceptualization, Methodology, Investigation, Data curation, Writing—review and editing; TA: Writing original draft as well as review and editing; IG: Writing original draft as well as review and editing; LS: Conceptualization, Methodology, Writing—review and editing; EH: Conceptualization, Methodology, Investigation, Writing—review and editing; DL: Conceptualization, Methodology, Investigation, writing—review and editing; KL: Software, Formal analysis, Writing original draft as well as review and editing, Visualization.

## Conflict of Interest

The authors declare that the research was conducted in the absence of any commercial or financial relationships that could be construed as a potential conflict of interest.
