# Supplementary material for: Perceived Risk and Protection From Infection and Depressive Symptoms Among Healthcare Workers in Mainland China and Hong Kong During COVID-19
Source: Front Psychiatry. 2020 Jul 15;11:686. doi: 10.3389/fpsyt.2020.00686 (PMC7378321; doi:10.3389/fpsyt.2020.00686)
Supplement: Supplementary file 1 [file DataSheet_1.docx]

**Data supplement**

Table of Contents

[Questionnaire 2](#_Toc38455096)

[Table 1 Adjusted* PHQ-9 score and screen-detected positive depression among nurses and physicians in Mainland China and Hong Kong 6](#_Toc38455097)

[Table 2 Adjusted* PHQ-9 score and screen-detected positive depression among 932 Chinese healthcare workers by the number of risk factors present 7](#_Toc38455098)

[Table 3 Adjusted prevalence of depression according to health beliefs among 932 Chinese healthcare workers 8](#_Toc38455099)

# Questionnaire

***Part 1: Background information***

1. Your gender:

○ Male ○ Female

2. Your profession:

○ Doctor ○ Nurse ○ Technician ○ Logistical ○ Other

3. The department / nursing unit where you work

_________________________________

4. Educational level:

○ College ○ Undergraduate ○ Master ○ Doctorate

5. Marital status:

○ Single ○ In a relationship ○ Married

○ Divorced / Separated ○ Widowed ○ Other _________________

6. How many years have you been engaged in a hospital-related work?

_________________________________

7. Have you ever been in contact with a confirmed or suspected case of COVID-19?

○ Yes ○ No

8. On average, how many patients with infectious respiratory diseases are you in contact with during your work every day?

_________________________________

***Part 2: The practice of using face mask***

9. Which type of masks is currently provided by your department?

○ Disposable medical masks ○ Disposable medical surgical masks

○ KN95 / N95 and above respirators ○ N95 medical grade respirators

10. What are the requirements for providing the masks in your department?

○ Disposable medical masks, requirements being: _________________

○ Disposable medical surgical masks, requirements being: _________________

○ KN95 / N95 and above respirators, requirements being: _________________

○ N95 medical grade respirators , requirements being: _________________

11. During the COVID-19 epidemic, which type of masks do you most prefer to wear when working in the hospital?

○ Disposable medical masks ○ Disposable medical surgical masks

○ KN95 / N95 and above respirators ○ N95 medical grade respirators

12. Do you think the personal protective equipment provided by the hospital is adequate during the COVID-19 outbreak?

○ Not at all ○ Sometimes ○ Basically adequate ○ Very adequate

13. During the COVID-19 epidemic, are you satisfied with the infection control training provided by the hospital?

○ Very unsatisfied ○ Unsatisfied ○ Basically satisfied ○ Very satisfied

***Part 3: Health awareness in using masks***

14. Do you feel vulnerable to contracting the novel coronavirus?

○ Not at all ○ Slightly ○ Very ○ Extremely

15. Did you know or had close contact with any individuals infected with the novel coronavirus?

○ Yes ○ No

16. Did you recently have symptoms similar to novel coronavirus infection (such as sore throat, cough, fever, muscle ache, and shortness of breath)?

○ Yes ○ No

17. To what extent are you fearful of contracting the novel coronavirus?

○ Not at all ○ To a very small extent ○ To a moderate extent ○ To a very great extent

18. To what extent are you worried that your current place of residence would become an epidemic area because of the widespread disease outbreak in the community?

○ Not at all ○ To a very small extent ○ To a moderate extent ○ To a very great extent

19. To what extent do you agree that wearing a face mask could prevent contracting and spreading the novel coronavirus?

○ Not at all ○ To a very small extent ○ To a moderate extent ○ To a very great extent

20. How difficult is it for you to get a face mask?

○ Not at all ○ To a very small extent ○ To a moderate extent ○ To a very great extent

21. What is the level of discomfort when wearing a face mask?

○ Not at all ○ To a very small extent ○ To a moderate extent ○ To a very great extent

22. To what extent do you think the local government encourages you to wear a face mask?

○ Not at all ○ To a very small extent ○ To a moderate extent ○ To a very great extent

23. To what extent do you think your family and/or colleagues or friends encouraged you to wear a face mask?

○ Not at all ○ To a very small extent ○ To a moderate extent ○ To a very great extent

24. Do you think you have adequate knowledge about COVID-19?

○ Not at all ○ To a very small extent ○ To a moderate extent ○ To a very great extent

25. Does the local health authority provide adequate knowledge of the novel coronavirus?

○ Not at all ○ To a very small extent ○ To a moderate extent ○ To a very great extent

26. To what extent do you believe you are able to wear a face mask properly?

○ Not at all ○ To a very small extent ○ To a moderate extent ○ To a very great extent

***Part 4：Mental Health***

Over the last 2 weeks, how often have you been bothered by any of the following problems?

27. Little interest or pleasure in doing things

○ Not at all ○ Several days ○ More than half the days ○ Nearly every day

28. Feeling down, depressed, or hopeless

○ Not at all ○ Several days ○ More than half the days ○ Nearly every day

29. Trouble falling or staying asleep, or sleeping too much

○ Not at all ○ Several days ○ More than half the days ○ Nearly every day

30. Feeling tired or having little energy

○ Not at all ○ Several days ○ More than half the days ○ Nearly every day

31. Poor appetite or overeating

○ Not at all ○ Several days ○ More than half the days ○ Nearly every day

32. Feeling bad about yourself or that you are a failure or have let yourself or your family down

○ Not at all ○ Several days ○ More than half the days ○ Nearly every day

33. Trouble concentrating on things, such as reading the newspaper or watching television

○ Not at all ○ Several days ○ More than half the days ○ Nearly every day

34. Moving or speaking so slowly that other people could have noticed. Or the opposite being so fidgety or restless that you have been moving around a lot more than usual

○ Not at all ○ Several days ○ More than half the days ○ Nearly every day

35. Thoughts that you would be better off dead, or of hurting yourself

○ Not at all ○ Several days ○ More than half the days ○ Nearly every day

# Table 1 Adjusted* PHQ-9 score and screen-detected positive depression among nurses and physicians in Mainland China and Hong Kong

|  | Guangdong | Hubei | Hong Kong |
| --- | --- | --- | --- |
| Mean (95% CI) PHQ-9 score |  |  |  |
| Nurses | 4.2 (3.4-5.0) | 5.4 (4.4-6.4) | 10.4 (9.7-11.1) |
| Physicians | 2.9 (2.1-3.8) | 4.1 (3.0-5.2) | 9.2 (7.8-10.5) |
| PHQ-9 positive |  |  |  |
| Nurses | 13.1 (8.6-17.6) | 14.8 (7.9-21.6) | 50.1 (43.7-56.4) |
| Physicians | 10.1 (6.2-17.0) | 12.6 (5.3-17.5) | 42.7 (27.7-57.8) |
| Moderately severe/severe depression | | | |
| Nurses | 4.1 (1.6-6.5) | 5.6 (1.4-9.7) | 23.6 (18.2-29.0) |
| Physicians | 1.9 (0.3-3.6) | 2.4 (-0.1-4.9) | 12.6 (1.6-23.6) |

*Adjusted for sex, educational level, marital status, location, profession, ward/unit, work experience, close contact with confirmed or suspected COVID-19 cases, personal protective equipment provision, infection control training, and presence of COVID-19-like symptoms

# Table 2 Adjusted* PHQ-9 score and screen-detected positive depression among 932 Chinese healthcare workers by the number of risk factors present

|  | Guangdong | Hubei | Hong Kong |
| --- | --- | --- | --- |
| *None of the 4 risk factors* |  |  |  |
| n | 273 | 22 | 24 |
| PHQ-9 score |  |  |  |
| Mean (SD) | 3.8 (4.3) | 5.8 (5.1) | 4.8 (3.9) |
| Adjusted*; mean (95% CI) | 5.8 (4.9-6.8) | 6.7 (5.6-7.8) | 8.5 (7.5-9.5) |
| PHQ-9 positive; % (95% CI) |  |  |  |
| Unadjusted | 7.7 (5.1-11.5) | 9.1 (2.2-30.7) | 8.3 (2.0-28.6) |
| Adjusted* | 7.0 (4.2-9.7) | 5.6 (1.5-9.6) | 18.7 (9.8-27.5) |
| *Having 1* | | | |
| n | 159 | 93 | 40 |
| PHQ-9 score |  |  |  |
| Mean (SD) | 5.4 (5.2) | 5.2 (4.7) | 7.9 (5.6) |
| Adjusted*; mean (95% CI) | 6.8 (5.9-7.8) | 7.7 (6.3-9.1) | 9.5 (8.6-10.4) |
| PHQ-9 positive; % (95% CI) |  |  |  |
| Unadjusted | 18.2 (13.0-25.1) | 16.1 (9.9-25.1) | 32.5 (19.8-48.5) |
| Adjusted* | 17.1 (11.5-22.6) | 14.0 (7.0-21.0) | 38.7 (26.6-50.8) |
| *Having 2* |  |  |  |
| n | 68 | 29 | 98 |
| PHQ-9 score |  |  |  |
| Mean (SD) | 5.5 (4.8) | 6.0 (4.4) | 10.4 (6.1) |
| Adjusted*; mean (95% CI) | 8.5 (7.2-9.9) | 9.4 (7.7-11.1) | 11.2 (10.4-12.1) |
| PHQ-9 positive; % (95% CI) |  |  |  |
| Unadjusted | 16.2 (9.1-27.0) | 17.2 (7.2-35.7) | 52.0 (42.1-61.8) |
| Adjusted* | 22.5 (15.0-30.0) | 18.7 (8.4-28.9) | 47.2 (37.7-56.6) |
| *Having 3 or 4* |  |  |  |
| n | 10 | 2 | 114 |
| PHQ-9 score |  |  |  |
| Mean (SD) | 8.4 (5.8) | 7.0 (2.8) | 12.6 (6.3) |
| Adjusted*; mean (95% CI) | 9.7 (8.0-11.4) | 10.5 (8.6-12.4) | 12.4 (10.9-13.9) |
| PHQ-9 positive; % (95% CI) |  |  |  |
| Unadjusted | 50.0 (21.3-78.7) | - | 64.0 (54.8-72.3) |
| Adjusted* | 37.3 (23.2-51.3) | 32.0 (14.3-49.7) | 64.6 (55.9-73.4) |

*Adjusted for sex, educational level, marital status, location, profession, ward/unit, work experience, number of risk factors

# Table 3 Adjusted prevalence of depression according to health beliefs among 932 Chinese healthcare workers

|  | Guangdong | Hubei | Hong Kong |
| --- | --- | --- | --- |
|  | Prevalence % (95% CI) | | |
| *Feeling susceptible to COVID-19 infection* | | | |
| Not at all/to a very small extent | 7.9 (5.3-10.5) | 9.6 (4.1-15.1) | 20.8 (12.5-29.0) |
| To a moderate extent | 24.4 (15.9-32.9) | 28.6 (13.9-43.3) | 49.7 (39.6-59.8) |
| To a very great extent | 31.1 (20.1-42.1) | 35.8 (18.8-52.9) | 58.0 (45.0-71.0) |
| *Fearful of contracting COVID-19* |  |  |  |
| Not at all/to a very small extent | 7.6 (5.0-10.3) | 9.3 (4.0-14.6) | 20.2 (11.4-29.0) |
| To a moderate extent | 19.3 (12.2-26.3) | 22.8 (10.6-35.1) | 42.2 (31.6-52.8) |
| To a very great extent | 28.8 (18.9-38.7) | 33.4 (16.0-50.7) | 55.3 (45.3-65.3) |
| *Wearing face mask could prevent contracting COVID-19* | | | |
| Not at all/to a very small extent | 11.3 (3.1-19.4) | 13.6 (2.1-25.0) | 27.9 (10.4-45.5) |
| To a moderate extent | 16.5 (11.4-21.7) | 19.7 (8.7-30.7) | 37.7 (25.8-49.7) |
| To a very great extent | 11.3 (7.4-15.2) | 13.6 (6.2-21.0) | 28.1 (19.4-36.7) |
| *Difficult to get face masks* |  |  |  |
| Not at all/to a very small extent | 8.2 (5.3-11.1) | 9.9 (4.6-15.3) | 21.4 (12.9-30.0) |
| To a moderate extent | 18.9 (12.1-25.8) | 22.4 (.2-35.6) | 41.7 (30.4-52.9) |
| To a very great extent | 28.7 (19.4-38.1) | 33.3 (16.1-50.5) | 55.2 (43.9-66.5) |
| *Encouraged by family and peers to wear face mask* | | | |
| Not at all/to a very small extent | 23.7 (12.2-35.2) | 27.8 (11.2-44.4) | 48.7 (31.4-66.1) |
| To a moderate extent | 15.2 (9.6-20.8) | 18.1 (7.4-28.9) | 35.4 (22.8-48.0) |
| To a very great extent | 11.5 (7.8-15.3) | 13.9 (6.3-21.5) | 28.5 (19.7-37.3) |
| *Having adequate knowledge about COVID-19* | | | |
| Not at all/to a very small extent | 18.2 (9.3-27.0) | 21.6 (7.5-35.7) | 40.5 (28.4-52.5) |
| To a moderate extent | 15.2 (10.6-19.8) | 18.2 (8.0-28.3) | 35.4 (25.7-45.1) |
| To a very great extent | 9.3 (5.6-13.0) | 11.3 (5.1-17.5) | 24.0 (13.6-34.3) |

* Adjusted for sex, educational level, marital status, location, profession, ward/unit, work experience, number of risk factors and mutual adjustment of the health belief variables
